# Knowledge, attitude, and practice regarding the prevention of osteoporosis among middle and old-aged women of Kirtipur Municipality, Nepal

**DOI:** 10.1371/journal.pone.0312738

**Published:** 2024-10-25

**Authors:** Surakshya Khanal, Manish Rajbanshi, Aashish Rana, Subash Wagle, Richa Aryal, Dinesh Raj Neupane, Buna Bhandari

**Affiliations:** 1 Central Department of Public Health, Institute of Medicine, Tribhuvan University, Kathmandu, Nepal; 2 Padma Kanya Multiple Campus, Tribhuvan University, Kathmandu, Nepal; 3 Department of Global Health and Population, Harvard T.H Chan School of Public Health, Boston, Massachusetts, United States of America; King Abdulaziz University Faculty of Medicine, SAUDI ARABIA

## Abstract

Osteoporosis is a major public health problem that can lead to physical disability, working performance limitations, decreased self-sufficiency, and increased hospitalization and mortality rates. People are unaware of osteoporosis, and it is often undiagnosed until fractures occur. Limited studies have been conducted to determine the knowledge, attitude, and practice regarding osteoporosis among middle-aged women in Nepal. This study aimed to determine existing knowledge, attitude, and practice towards osteoporosis among middle and old-aged women in Nepal. A cross-sectional study was conducted among the selected wards of Kirtipur Municipality to recruit 405 participants. Participants were selected using a stratified random sampling technique. Face-to-face interviews using a structured questionnaire were performed to collect the data. Frequencies, percentages, mean, and standard deviation were used to describe the characteristics of participants. Multivariate logistic regression was used to determine the factors associated with knowledge, attitude, and practice regarding osteoporosis. Pearson’s correlation coefficient was used to determine the correlation between knowledge, attitude, and practice regarding osteoporosis. The mean ± SD age of the participants was 46.2 ±9.1 years. Nearly half of the participants (48.8%) had good knowledge, while 57.7% and 51.8% had positive attitudes and good practices regarding osteoporosis, respectively. The occupation and income of the participants were statistically significant and associated with the knowledge level. Meanwhile, age, ethnicity, education, occupation, and monthly household income were associated with attitude level. With a good practice level, ethnicity, family type, and education were statistically significant. The knowledge-attitude (r_ka_ = 0.093, p < 0.05), attitude-practice (r_ap_ = 0.171, p < 0.001), and knowledge-practice (r_kp_ = 0.274, p < 0.001) for osteoporosis were positively correlated. The study found that still around half of the middle and old-aged women had poor knowledge, negative attitudes, and poor practices regarding osteoporosis. Moreover, it highlighted inadequate dietary practices, such as low consumption of milk, vegetables, fruits, and calcium supplements among women, indicates are at greater risk of osteoporosis. The study emphasized the need for community-based awareness programs for the target population such as housemakers, and lower-income groups, to prevent osteoporosis among women.

## Introduction

Osteoporosis is a disease characterized by low bone mass and microarchitecture deterioration of bone tissue, leading to enhanced bone fragility and a consequent risk of fracture [1]. Due to the absence of signs and symptoms until a fracture, it is frequently referred to as a silent disease. Osteoporosis affects both sexes and all ages, but it is two to three times more common among women than men [2]. The entire population irrespective of age is at risk of osteoporosis, moreover, old age and post-menopausal women are more susceptible to the development of osteoporosis [3]. Osteoporosis and related fractures can lead to physical disability, working performance limitations, decreased self-sufficiency, and increased hospitalization and mortality rates [2].

The World Health Organization (WHO) estimated that osteoporosis affects 200 million women and causes more than 8.9 million fractures each year globally by 2050 [4, 5]. Among them, more than 50% of all osteoporotic fractures will occur in Asian countries [6]. One in three women and one in five men over the age of 50 experience osteoporotic fractures in their lifetime [2]. Osteoporosis and related fractures might be responsible for 5.91 million dollars by 2035, with a total medical cost of 25.43 billion dollars by 2050 [7]. The situation will further be exacerbated, especially among the population that resides in rural areas where osteoporosis is often left undiagnosed and untreated.

Previous studies reported that the rising incidence of osteoporosis is attributed to poor health literacy, inadequate nutritional behaviour (such as low intake of calcium, smoking, and drinking alcohol), lack of physical exercise, low body weight, lack of access to healthcare services, and eating and food choices of women [5, 8, 9]. In addition, the burden of osteoporosis is still neglected at the community level, people are unaware of the connection between osteoporosis, lifestyle, and nutrition behaviors [10].

A study conducted in 2019 in people aged 50 years and above in hospitals in Kathmandu showed the prevalence of osteoporosis at 37.3%, osteopenia at 38.5%, and normal bone mineral density (BMD) at 24.2% [11]. Similarly, a study conducted in 2019 at different hospitals in central Nepal among healthy males and females above 20 years showed that the prevalence of osteoporosis and osteopenia was 22.3% and 60.6% respectively [12]. Previously, a study conducted in western Nepal among middle-aged and elderly adults showed a very high prevalence of 73.68% of Vitamin D deficiency, revealing that females were 5.29% more deficient than males, due to the result of inadequate sun exposure, increasing urbanization, and poor outdoor activity [13].

Many studies suggested that preventing osteoporosis and its consequences can greatly reduce the health system’s expenditures [9]. Several studies reported that the prevalence of osteoporosis is high in Nepal and varies from 8.2% to 22.4% [9–11]. In Nepal, people are unaware of osteoporosis, and it is often undiagnosed until fractures occur [5]. Limited studies have been conducted to determine the knowledge, attitude, and practice regarding osteoporosis among middle-aged women, meanwhile, some studies focused on the hospital setting and older adults [14]. Hence, this study aimed to determine the knowledge, attitude, and practice (KAP) regarding osteoporosis and its associated factors among middle and old-aged women of Kirtipur Municipality of Kathmandu district in Nepal.

## Materials and methods

### Study design and settings

A community-based cross-sectional study was conducted among the selected wards of Kirtipur Municipality (KM). This municipality is located in Kathmandu district, Nepal. It is a semi-urban and ancient city of Kathmandu district with an area of 14.76 sq. km [15]. This municipality is divided into 19 wards. The total population of this Municipality is 81,578 with a population density of 5527/km^2^ [16]. The literacy rate of this municipality was 88.8%, according to the latest census in 2021 [17]. This municipality consists of 13 governmental and 6 non-government health institutions [18].

### Study population

This study included women aged 30 years or above who had lived for at least six months in Kirtipur Municipality. Women who had hearing problems, speech problems and were unable to communicate were excluded from this study. Also, pregnant women, lactating mothers and participants who were unwilling to participate were not taken in this study.

### Sample size and sampling technique

The sample size was calculated using the Cochrane proportionate formula (n = Z^2^pq/d^2^) [19]. The proportion of having good knowledge of osteoporosis (p = 61.3%) was obtained from a similar study Neupane et al. 2023 [20].

Assuming a 5% allowable error, 95% Confidence Interval (CI), and a 10% non-response rate, the minimum number of required sample sizes was 405 for this study. Out of 19 wards in Kirtipur Municipality, 3 wards were selected randomly using the lottery method as the study site, i.e., Ward 1, Ward 3, and Ward 8. Out of the three selected wards, there was a total of 4658 households consisting of 1915 households in Ward 1, 1414 in Ward 3, and 1359 in Ward 8.

Then, proportionate sampling was performed to determine the number of samples for each ward based on the number of households. The number of samples for each selected household was Ward 1 (n_1_ = 166), Ward 3 (n_3_ = 123), and Ward 8 (n_8_ = 116). A systematic random sampling technique, i.e., (N/n)^th^ household was followed to select participants from the selected household. In the absence of eligible participants in the selected household, the nearby household was preferred to choose the participants. In the case of more than one eligible participant in the same household, the eldest women were considered for this study.

### Data collection

The face-to-face interview was conducted to collect the data from the selected participants. The data was collected using a structured questionnaire. The first author (SK) was responsible for conducting the interview. Each interview took around 30–35 minutes to complete. The data collection was carried out between January 30 to February 28, 2021.

### Tools and measures

The knowledge regarding osteoporosis was determined by using the OKAT tool [3, 21]. Questions related to attitude and practice regarding osteoporosis were adopted from similar studies [3, 20, 22]. A Nepali-language translated tool was used and pretested among 10% of the actual sample size (n = 40) in the nearest municipality to KM to check its internal consistency. The results showed that the Cronbach alpha coefficients of KAP domains were 0.82, 0.78, and 0.80, respectively.

The questionnaire had four parts. The first included social and demographical characteristics of participants, such as age (in years), ethnicity, religion, family type, educational level, monthly household income (in NRs.), and occupation. The second part consisted of knowledge questions and was assessed by the OKAT tool [21]. It included signs/symptoms, causes and risk factors, preventive measures, and complications of osteoporosis. Knowledge was evaluated by assigning scores ‘1’ for the positive responses and ‘0’ for the negative responses. Then, the mean score was obtained from the participant’s response. The values equal to the mean or above (score ≥39) were as good knowledge, and values below the mean score (score<39) were categorized as poor knowledge regarding osteoporosis [20].

The third part included questions regarding attitudes toward osteoporosis, risk factors for osteoporosis, the seriousness of osteoporosis, complications due to osteoporosis, and preventive measures for osteoporosis. Participants were asked to rate their attitude on osteoporosis using a five-point Likert scale. It was measured by assigning scores 1, 2, 3, 4, and 5 corresponding to the following responses: Strongly Disagree, Disagree, Not Sure, Agree, Strongly Agree. Then, participants’ scores equal to the mean or above (score ≥ 40) were categorized as positive attitudes, and values below the mean score (score <40) as negative attitudes [3].

The fourth section focused on questions related to preventive practices for osteoporosis. It included topics such as physical exercise, dietary patterns, avoidance of smoking and alcohol, and early screening for osteoporosis prevention. Each practice item was responded to in terms of everyday, regular, rarely, and never scale. Then, each participant’s response was assigned a score of 1, 2, 3, or 4 to the corresponding reactions every day, regularly, rarely, and never respectively. The participants’ scores equal to the mean or above (score ≥ 28) were regarded as “good practice”, and values below the mean score (score < 28) as “poor practice” towards osteoporosis prevention [23].

### Data management

Collected data were systematically entered, filtered, coded, and cross-checked in Epi-Data version 3.1 software. Data analysis was performed using Statistical Package for the Social Sciences (SPSS) version 20.0 (IBM). The participants’ socio-demographic characteristics were described using frequencies, percentages, mean and standard deviation. Multivariate regression analysis determined the factors associated with knowledge, attitude, and practice of osteoporosis. Pearson correlation coefficient was used to assess the relationship between knowledge-attitude, attitude-practice, and knowledge-practice. The statistical significance was set at a p-value <0.05.

### Ethical statement

This study was reviewed and approved by the Institutional Review Board (IRB) of the Institute of Medicine (IOM), Tribhuvan University, Nepal [Reference no: 203(6–11)E^2^077/078]. Before the start of data collection, a letter of support was obtained from the Municipal Office and Ward Office. After outlining the purpose of the study, both verbal and written consent was obtained from each participant.

## Results

### Socio-economic and demographic characteristics

A total of 405 individuals participated in this study. The mean (± S.D) age was 46.2±9.07 years. Most participants were Janajati (67.4%) in ethnicity. One-fourth (24.1%) had not received formal education. The maximum number of participants (46.7%) had a monthly household income of NRs 21,000- NRs 40,000 (USD 157.29—USD 299.59 USD) (Table 1).

**Table 1:** Individual characteristics of the participants.

Characteristics	Frequency (n)	Percentage (%)
**Age (in completed years)**		
Mean± S.D. (46.2 ±9.07)		
30–40	137	33.9
41–50	146	36.0
Above 50	122	30.1
**Ethnicity**		
Janajati	273	67.4
Brahmin/Chhetri	57	14.1
Dalit	45	11.1
Madhesi	30	7.4
**Religion**		
Hindu	322	79.5
Buddhist	63	15.6
Muslim	20	4.9
**Type of family**		
Nuclear	250	61.7
Joint/extended	155	38.3
**Education**		
No formal education	98	24.2
Primary level	142	35.1
Secondary level	72	17.8
Higher secondary and above level	93	22.9
**Monthly household income** (In NRs)		
Below or equal to 20000 (≤USD 149.8)	156	38.5
21,000–40,000 (USD 157.29—USD 299.59)	189	46.7
≥ 41,000 (≥USD 307.08)	60	14.8
**Occupation**		
Job	150	37.0
House maker	148	36.6
Others (*business*, *agriculture and labor*)	133	26.4

### Source of healthcare information

**Fig 1:**
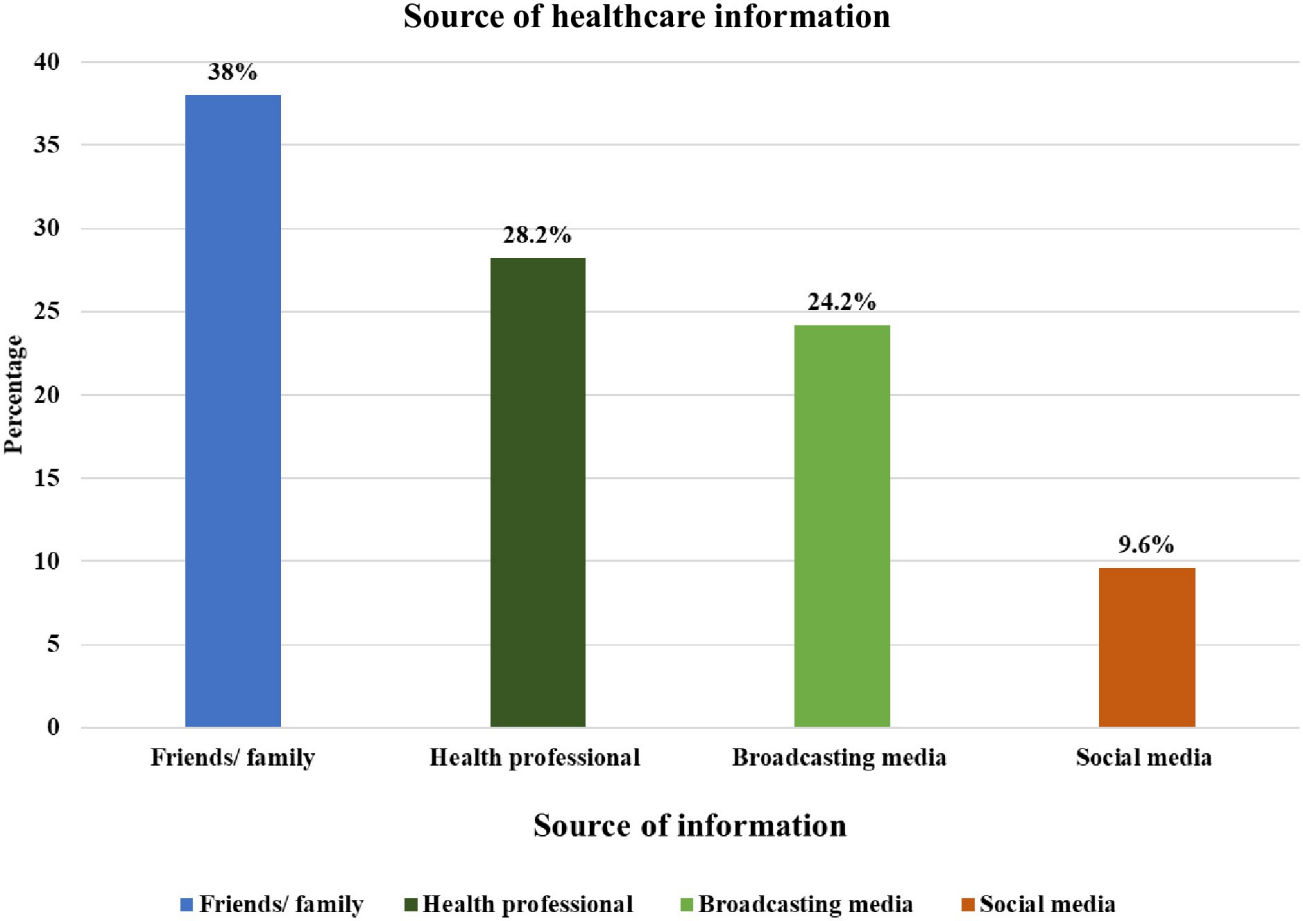
Source of healthcare information.

Fig 1 depicts that friends/family (38%) and health professionals (28.2%) were the most common sources for accessing healthcare information.

### Knowledge towards osteoporosis

Around three-fourths (76.5%) of the participants knew that osteoporosis leads to an increased risk of bone fracture. Only one-third (33.8%) knew that cigarette smoking can contribute to osteoporosis. Nearly three-fourths (72.8%) of them correctly identified that women aged 80 years or more are at a greater risk of osteoporosis. Around 80% of the participants were aware that physical activity prevents osteoporosis. More than half (52.8%) knew that sardines and broccoli were good calcium sources that prevent osteoporosis. Most participants (61%) correctly identified that calcium supplements can prevent bone loss alone (Table 2).

**Table 2:** Knowledge regarding osteoporosis among the study participants.

	Knowledge items	Correct response n (%)
Q1	Osteoporosis increases the risk of bone fracture.	310 (76.5)
Q2	Osteoporosis usually causes symptoms before fractures occur.	170 (42.0)
Q3	Having a higher peak bone mass at the end of childhood gives no protection against the development of osteoporosis in later life.	168 (41.5)
Q4	Osteoporosis is more common in men.	263 (64.9)
Q5	White women are at highest risk of fracture as compared to other races.	209 (51.6)
Q6	Cigarette smoking can contribute to osteoporosis.	137 (33.8)
Q7	A fall is just as important as low bone strength in causing fractures.	337 (83.2)
Q8	Women aged 80 years or more are at the greater risk of osteoporosis.	295 (72.8)
Q9	From age 50, most women can expect at least one fractures before they die.	269 (66.4)
Q10	Any type of physical activity is beneficial for osteoporosis.	324 (80.0)
Q11	It is easy to tell whether I am at risk of osteoporosis by my clinical risk factors.	280 (69.1)
Q12	Family history of osteoporosis strongly predisposes a person to osteoporosis.	293 (72.3)
Q13	An adequate calcium intake can be achieved from two glasses of milk a day.	307 (75.8)
Q14	Sardines and broccoli are good sources of calcium for people who cannot take dairy products.	214 (52.8)
Q15	Calcium supplements alone can prevent bone loss.	247 (61.0)
Q16	Alcohol in moderation has little effect on osteoporosis.	315 (77.8)
Q17	A high salt intake is a risk factor for osteoporosis.	140 (34.6)
Q18	There is a small amount of bone loss in the ten years following the onset of menopause.	143 (35.3)
Q19	Hormone therapy prevents further bone loss at any age after menopause.	254 (62.7)
Q20	There are no effective treatments for osteoporosis available in Nepal.	47 (22.2)

### Factors associated with knowledge regarding osteoporosis

Table 3 shows that the participants who were housemakers had lower odds of having good knowledge regarding osteoporosis (AOR = 0.08, CI: 0.04–0.20). Participants with higher monthly household income (AOR = 4.8, CI: 1.8–13.3) were significantly associated with good knowledge regarding osteoporosis.

**Table 3:** Factors associated with knowledge regarding osteoporosis among the study participants.

Characteristics	Knowledge			P-value		P-value
	Good n (%)	Poor n (%)	COR, 95% CI		AOR, 95% CI	
**Level**	198 (48.8)	207 (51.2)				
**Age**						
Below 45	92 (49.5)	94 (50.5)	1.04 (0.7–1.5)	0.81	1.0 (0.6–1.7)	0.901
45 or above	106 (48.4)	113 (51.6)			ref	
**Ethnicity**						
Janajati	99 (36.3)	174 (63.7)	0.19 (0.1–0.3)	0.00	0.6 (0.4–1.2)	0.211
Others	99 (75.0)	33 (25.0)			ref	
**Religion**						
Hindu	18 (90.0)	2 (10.0)	10.2 (2.3–44.7)	0.02	3.0 (0.6–14.1)	0.215
Others	189 (46.8)	205 (53.2)			ref	
**Family type**						
Nuclear	134 (53.6)	116 (46.4)	0.6 (0.4–0.9)	0.019	0.7 (0.4–1.3)	0.304
Joint/extended	64 (41.3)	91 (58.7)			ref	
**Education**						
Formal education	184 (59.9)	123 (40.1)	8.9 (4.8–16.5)	0.00	1.2 (0.5–2.8)	0.70
No formal education	14 (14.3)	84 (85.7)			ref	
**Occupation**						
Housemaker	19 (12.8)	129 (87.2)	0.06 (0.0–0.1)	0.00	**0.08 (0.0–0.2)**	**<0.001**
Others	179 (69.6)	78 (30.4)			ref	
**Monthly household income (in NRs)**						
Below 20,000	8(14.8)	46 (85.2)	6.7 (3.1–14.8)	0.00	**4.8 (1.8–13.3)**	**0.002**
Above 20,000	190 (54.1)	161(45.9)			ref	

ref: reference category

### Attitude towards osteoporosis

**Fig 2:**
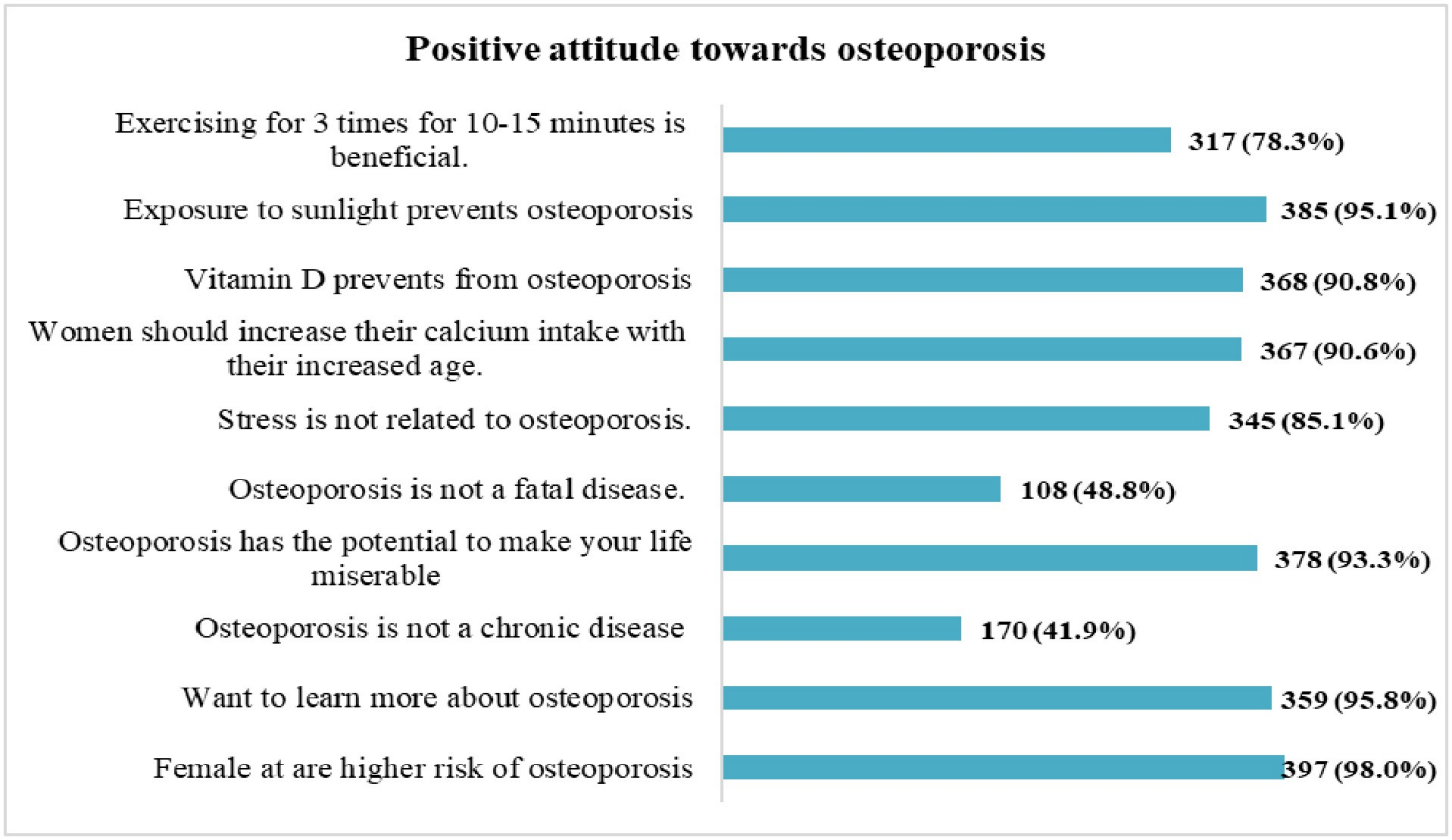
Attitude regarding osteoporosis among the study participants.

Fig 2 shows that almost all of the participants (98.02%) believed that females are at a higher risk of developing osteoporosis than males. Around 42% stated that osteoporosis is not a severe disease like cancer and diabetes. half (48.8%) believed osteoporosis is not fatal. Most participants responded that calcium (90.6%) and Vitamin D (90.8%) prevent osteoporosis.

### Factors associated with attitude regarding osteoporosis

Table 4 shows that age (AOR = 0.5, CI: 0.3–0.7), ethnicity (AOR = 0.5, CI: 0.3–0.9), educational level (AOR = 1.9, CI: 1.0–3.5), occupation (AOR = 1.7, CI: 1.0–3.1), and monthly household income (AOR = 2.0, CI: 1.0–4.0) were significantly associated with attitude regarding osteoporosis.

**Table 4:** Factors associated with attitude regarding osteoporosis among the study participants.

Characteristics	Attitude			P-value		P-value
	Positive n (%)	Negative n (%)	COR, 95% CI		AOR, 95% CI	
**Level**	234 (57.7)	171 (42.3)				
**Age**						
Below 45	92 (49.5)	94 (50.5)	0.5 (0.4–0.8)	0.002	**0.5 (0.3–0.7)**	**0.001**
45 or above	142 (64.8)	77 (35.2)			ref	
**Ethnicity**						
Janajati	145 (53.1)	128 (46.9)	0.5 (0.4–0.9)	0.007	**0.5 (0.3–0.9)**	**0.04**
Others	89 (67.4)	43 (32.6)			ref	
**Religion**						
Hindu	175 (54.3)	147 (45.7)	1.7 (0.7–4.7)	0.26	1.4 (0.5–3.8)	0.321
Others	59 (71.1)	24 (28.9)			ref	
**Family type**						
Nuclear	151 (60.4)	99 (39.6)	0.7 (0.5–1.1)	0.18	0.8 (0.5–1.2)	0.30
Joint/extended	83 (53.5)	72 (46.5)			ref	
**Education**						
Formal education	190 (61.9)	117 (38.1)	2.0 (1.2–3.1)	0.003	**1.9 (1.0–3.5)**	**0.03**
No formal education	44 (44.9)	54 (55.1)			ref	
**Occupation**						
Housemaker	82 (55.4)	66 (44.6)	0.8 (0.5–1.2)	0.467	**1.7 (1.0–3.1)**	**0.04**
Others	152 (59.1)	105 (40.9)			ref	
**Monthly household income (NRs)**						
≤20,000	22 (40.7)	32 (59.3)		0.008	ref	
>20,000	212 (60.4)	139 (39.6)	2.2 (1.2–3.9)		**2.0 (1.0–4.0)**	**0.03**

ref: reference category

### Practice towards osteoporosis

**Fig 3:**
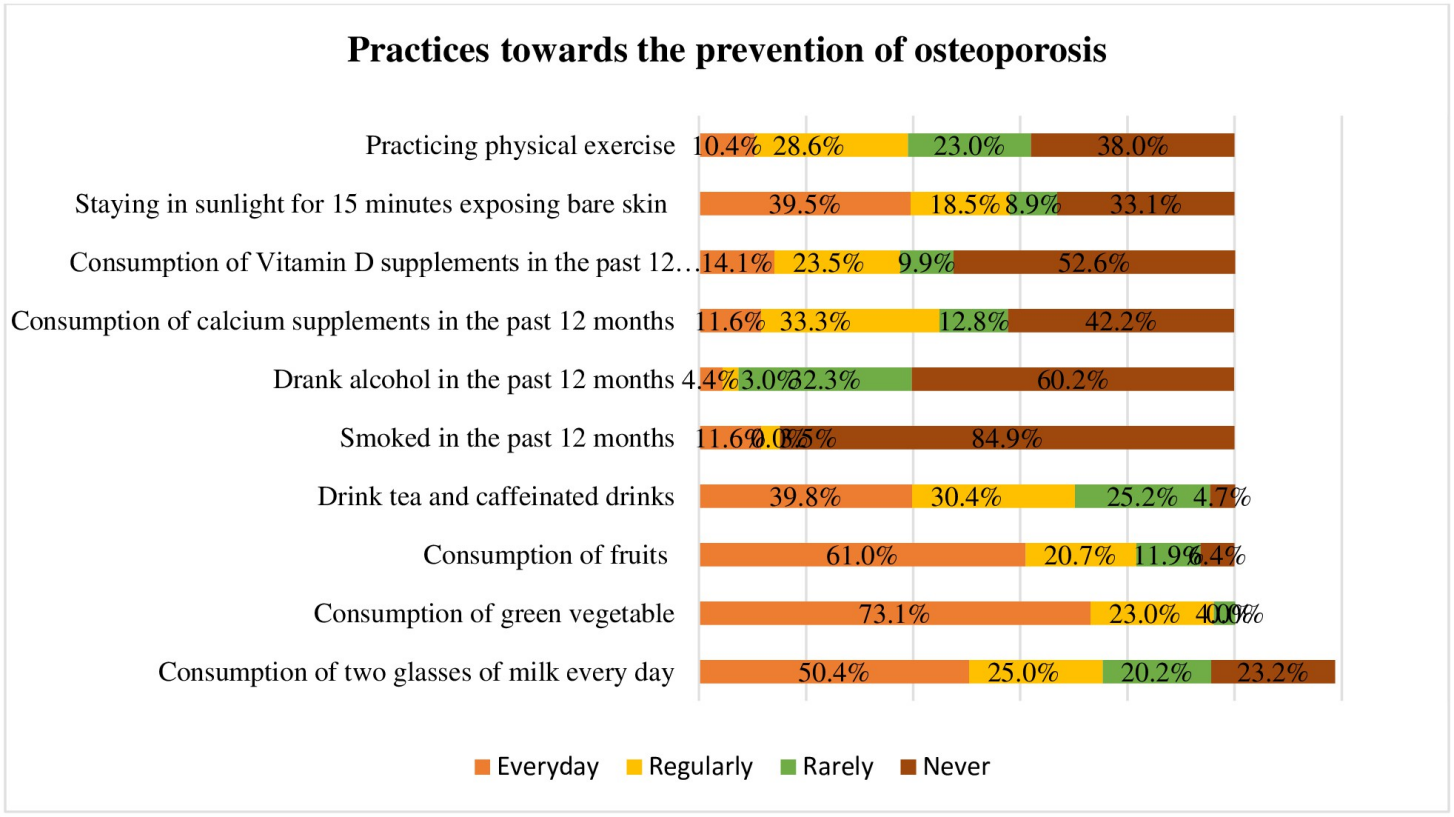
Practices towards the prevention of osteoporosis.

Fig 3 illustrates that half of the participants consumed two glasses of milk every day to prevent osteoporosis. Few participants regularly consumed green vegetables (23%) and fruits (20.7%). Most of the participants did not consume calcium (42.2%) and Vitamin D (52.6%) supplements in the past 12 months. Nearly one-third (28.6%) performed physical exercise regularly to avoid osteoporosis.

### Osteoporosis screening among study participants

**Fig 4:**
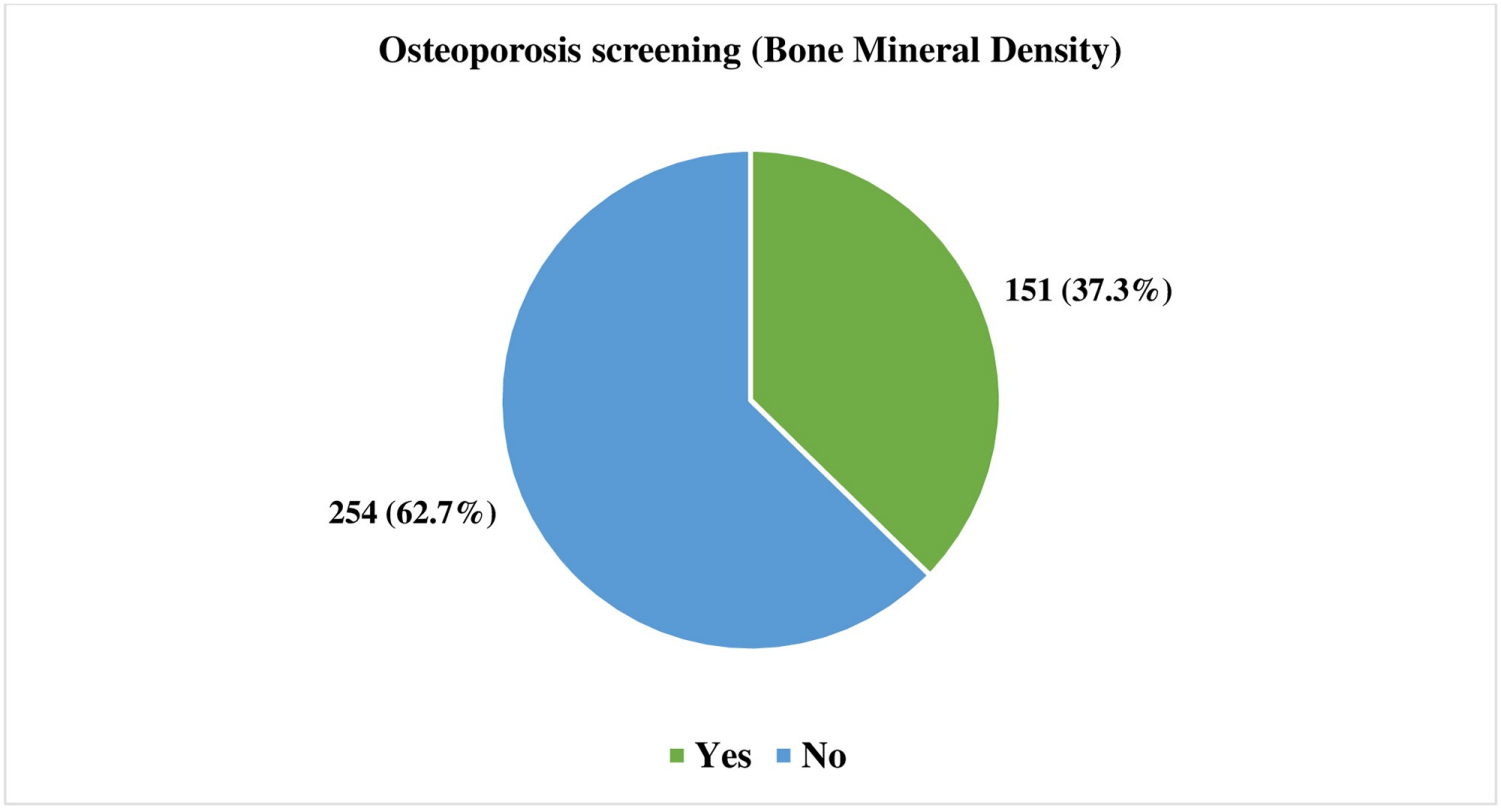
Osteoporosis screening among study participants.

This study found that 37.3% had done osteoporosis screening (Fig 4).

### Factors associated with practice regarding osteoporosis

Table 5 demonstrates that ethnicity (AOR = 0.43, CI: 0.25–0.74) and education (AOR = 3.23, CI: 1.67–6.24) of the participants were significantly associated with practice regarding osteoporosis.

**Table 5:** Factors associated with practice regarding osteoporosis among the study participants.

Characteristics	Practice			P-value		P-value
	Good n (%)	Poor n (%)	COR, 95% CI		AOR, 95% CI	
**Level**	210 (51.8)	195 (48.2)				
**Age**						
Below 45	94 (50.5)	92 (49.5)	0.91 (0.6–1.1)	0.690	0.90 (0.5–1.4)	0.64
45 or above	116 (53.0)	103 (47.0)			ref	
**Ethnicity**						
Janajati	158 (57.9)	115 (42.1)	0.28 (0.1–0.4)	0.00	**0.43 (0.2–0.7)**	**0.003**
Others	95 (72.0)	37 (28.0)			ref	
**Religion**						
Hindu	15 (75.0)	5 (25.0)	2.92 (1.0–8.2)	0.04	1.01 (0.3–3.2)	0.97
Others	195 (49.4)	190 (50.6)			ref	
**Family type**						
Nuclear	146 (58.4)	104 (41.6)	2 (1.3–3.0)	0.001	**1.64 (1.02–2.63)**	**0.04**
Joint/extended	64 (41.3)	91 (58.7)			ref	
**Education**						
Formal education	120 (39.1)	187 (60.9)	5.08 (3.0–8.5)	0.00	**3.23 (1.6–6.2)**	**0.00**
No formal education	23 (23.5)	75 (76.5)			ref	
**Occupation**						
Housemaker	55 (37.2)	93 (62.8)	0.39 (0.2–0.6)	0.00	1.20 (0.6–2.1)	0.52
Others	155 (60.3)	102 (39.7)			ref	
**Monthly household income (in NRs)**						
Below 20,000	15 (27.8)	39 (72.2)		0.00	ref	0.08
Above 20,000	195 (55.6)	156 (44.4)	3.25 (1.7–6.1)		1.90 (0.9–3.9)	

ref = reference category

### Correlation between knowledge, attitude, and practice regarding osteoporosis

This study showed knowledge was positively correlated with attitude (r_ka_ = 0.093, p<0.05) and practice (r_kp_ = 0.274, p<0.001) regarding osteoporosis. Similarly, attitude and practice were positively correlated (r_ap_ = 0.171, p<0.001) in this study (Table 6).

**Table 6:** Correlation between knowledge, attitude, and practice regarding osteoporosis among the study participants.

Characteristics	Pearson correlation coefficient (r)		
	Knowledge	Attitude	Practice
**Knowledge**	1	0.093*	0.274**
**Attitude**	0.093*	1	0.171**
**Practice**	0.274**	0.171**	1

* Statistically significant at the level of p-value less than 0.05

** Statistically significant at the level of p-value less than 0.001

## Discussion

The rising incidence of osteoporosis is attributed to poor health literacy, and inadequate nutritional behavior, such as low calcium intake, smoking, drinking alcohol, physical inactivity, low body weight, lack of access to healthcare services, and dietary choices. It is crucial to explore women’s awareness, beliefs, and practices regarding osteoporosis to implement effective control and preventive interventions at the community level. Hence, this study is carried out to determine the knowledge, attitude, and practice regarding osteoporosis among middle and old-aged women of Nepal.

This study found that almost half of the women had good knowledge regarding osteoporosis. This finding is similar to the study conducted in Kathmandu, Nepal [5]. In contrast, a similar study conducted in Malaysia (46%) [24] and India (27.6%) [25] showed that most women had a low level of knowledge. In this study, the majority of participants knew that osteoporosis increases the risk of bone fracture. This finding is consistent with the previous study in Nepal [3] and Saudi Arabia [26]. This might be due to most of the females in Nepal are involved as housemakers, so they are deprived of healthcare information from external sources.

The highest proportion of women knew that osteoporosis increases the risk of bone fracture, a similar finding to the previous study in Nepal [3], Saudi Arabia [26], and Malaysia [27]. Similarly, most participants were aware that calcium supplements prevent osteoporosis, a finding that coincides with a previous study in Nepal [5], Audi Arabia [26], and Turkey [28].

Similarly, this study found that women who were housemakers had lower odds of having knowledge and poor practices about osteoporosis than others. A similar finding was observed in the previous study in Nepal [20] and Pakistan [29]. Housemaker women are usually busy in kitchen practice, child-rearing, and agricultural fields, so they often have less exposure to health programs [20]. Meanwhile, those women engaged in occupations outside their homes have frequent exposure with their colleagues and friends, where they can share their health-relevant topics, including osteoporosis [20].

Women with high incomes were 4.8 times more likely to have good knowledge than others. This finding is similar to the studies done in Saudi Arabia [30] and Malaysia [31]. This might be because women of higher economic status have good platforms for higher education, health-seeking behaviour, and sources of health-related information. However, a similar study conducted in Malaysia showed no relationship between income and osteoporosis knowledge [24]. Similar to the other study findings, a majority of the participants in the current study perceived osteoporosis as less dangerous than other diseases. i.e., heart disease and cancer [32].

Comparatively with the relevant previous studies, this study demonstrated that the attitude of women towards osteoporosis varied with factors such as age, ethnicity, educational level, occupation, and income level. Women aged 45 and older were more likely to have a positive attitude towards osteoporosis than their counter groups. These findings are similar to the study conducted in Nepal [20], Singapore [33] and Saudi Arabia [30]. It is supported by previous studies that revealed older people are more vulnerable to health issues and frequently visit health institutions for health check-ups. This provides an opportunity to share health problems with health professionals, including chronic conditions such as osteoporosis.

This study revealed that the ethnicity of the participants influenced attitudes and practices towards osteoporosis. It is due to the discrepancy in health information based on ethnicity, cultural beliefs, or health literacy. These disparities may influence osteoporosis-related knowledge and attitudes among the different ethnicities.

Attitudes regarding osteoporosis were greatly influenced by formal education. Compared to those without formal education, participants with formal education were more likely to have a positive attitude toward osteoporosis. Similar results were observed in the previous studies [23, 27, 34]. Education raises health literacy and awareness and gives people the tools they need to manage health conditions such as osteoporosis [34]. People with higher education tend to have easier access to preventive care, health information, and healthcare resources, which can positively impact people’s attitudes toward health.

This result emphasizes how crucial educational interventions are for raising awareness and changing attitudes about osteoporosis. However, a study conducted in Malaysia among adults showed no association between education level and osteoporosis [35].

This study demonstrated that ethnicity influenced differences in preventive practices toward osteoporosis. This finding is consolidated by similar studies conducted in Saudi Arabia [36], Singapore [33] and India [25]. It is due to preventive and control practices towards any chronic conditions that are enhanced by access to healthcare information and services, educational status, and health-seeking behavior [25]. Meanwhile, these services are inequitably distributed in the community and vary with different groups of ethnicities, resulting in low preventive practices among lower ethnic groups.

Our study showed good preventive practices toward osteoporosis among individuals with higher education. This result is consistent with a study conducted in Malaysia [35]. In contrast, a study conducted in Saudi Arabia [23] and India [25] shows no association between education and practice about osteoporosis. People with higher education may have better access to reliable health information sources [35]. Educational attainment is often linked to health literacy, which is the ability to find, understand, and use health information to make informed decisions about health [35].

This study showed a significant positive correlation between knowledge, attitude, and practice towards osteoporosis. A similar pattern was established in the study conducted in India [25] and Malaysia [35]. This result is supported by a similar study conducted among Nepalese residents in Nepal which revealed that their gained knowledge and positive attitude shapes individuals’ preventive practice towards health problems. Increased knowledge about health issues, like osteoporosis or healthy eating, provides the essential understanding of why certain behaviors are important. People learn about risk factors, the benefits of healthy practices, and the potential consequences of inaction [37].

### Strengths and limitations

This study is one of the first community-based studies conducted among middle-aged women investigating knowledge, attitude, and practice regarding osteoporosis using standard methods.

Our findings should be assessed with caution. There might be some self-reported bias. Some influencing characteristics, such as culture and traditions, nutritional behaviour, environmental factors, and comorbid conditions, were not the scope of the study. Due to limited resources we could not validate the tool in Nepali language.

## Conclusion

This study demonstrated that a significant proportion of women were unaware of the risk factors, symptoms, and complications of osteoporosis. Women belonging to housemakers and lower economic status had poor knowledge about osteoporosis. Meanwhile, a large portion of women had poor food choices and practices, such as poor consumption of milk, vegetables and fruits, and calcium supplements.

This study concludes that knowledge is the key influence in changing an individual’s attitude and practice toward better health. It is recommended that intervention studies to improve knowledge, attitude, and practice toward osteoporosis should be planned and assessed their feasibility and effectiveness for scaling at a large scale at the community level.

## Supporting information

S1 Data (CSV)
